# Incorporating historical control information in ANCOVA models using the meta‐analytic‐predictive approach

**DOI:** 10.1002/jrsm.1561

**Published:** 2022-04-26

**Authors:** Hongchao Qi, Dimitris Rizopoulos, Joost van Rosmalen

**Affiliations:** ^1^ Department of Biostatistics Erasmus University Medical Center Rotterdam the Netherlands; ^2^ Department of Epidemiology Erasmus University Medical Center Rotterdam the Netherlands

**Keywords:** analysis of covariance (ANCOVA), Bayesian statistics, historical borrowing, meta‐analytic‐predictive (MAP)

## Abstract

The meta‐analytic‐predictive (MAP) approach is a Bayesian meta‐analytic method to synthesize and incorporate information from historical controls in the analysis of a new trial. Classically, only a single parameter, typically the intercept or rate, is assumed to vary across studies, which may not be realistic in more complex models. Analysis of covariance (ANCOVA) is often used to analyze trials with a pretest–posttest design, where both the intercept and the baseline effect (coefficient of the outcome at baseline) affect the estimated treatment effect. We extended the MAP approach to ANCOVA, to allow for variation in the intercept and the baseline effect across studies, and possibly also correlation between these parameters. The method was illustrated using data from the Alzheimer's Disease Cooperative Study (ADCS) and assessed with a simulation study. In the ADCS data, the proposed multivariate MAP approach yielded a prior effective sample size of 79 and 58 for the intercept and the baseline effect respectively and reduced the posterior standard deviation of the treatment effect by 12.6%. The result was robust to the choice of prior for the between‐study variation. In the simulations, the proposed approach yielded power gains with a good control of the type I error rate. Ignoring the between‐study correlation of the parameters or assuming no variation in the baseline effect generally led to less power gain. In conclusion, the MAP approach can be extended to a multivariate version for ANCOVA, which may improve the estimation of the treatment effect.

**Highlights**

**What is already known**

- The MAP approach is a Bayesian method based on the meta‐analytic methodology to synthesize and incorporate information from historical controls in the design and analysis of a new trial.
- Previous implementations only borrowed information of one parameter from historical trials, which may not be appropriate in complex models.

**What is new**

- The study extends the MAP approach to synthesize and incorporate information of multiple parameters using the ANCOVA model as a motivating example.
- The proposed approach leads to better inference for the parameter of interest, i.e. improved precision, higher statistical power.

Potential impact for *Research Synthesis Methods* readers outside the authors' field

- The extension could be implemented in other models with multiple parameters, for example, the model for diagnostic test studies with two parameters of interest (sensitivity, specificity).
- The proposed approach could also be used in the design and analysis of trials/experiments in other fields, for example, education, psychology, with a pretest–posttest design when comparable historical controls arms are available.

## 1 INTRODUCTION

In the design and analysis of clinical trials, information from control arms of previous clinical trials with a similar design is often available. Borrowing the historical information is appealing because it may yield more accurate estimate of the parameter of interest, increased statistical power, and reduced sample size requirements. To properly incorporate the information from historical control arms, several historical borrowing methods have been proposed.^1^ A popular method is the meta‐analytic‐predictive (MAP) approach, which assumes that the parameter of interest is exchangeable across studies (i.e., drawn from a common normal distribution).

The MAP approach is closely related to meta‐analysis. In the MAP approach, first a Bayesian random effects meta‐analysis of the historical data (only control arms of these studies) is performed, that is, without using observed data of the new trial. Whereas in an ordinary meta‐analysis, the population mean parameter would be the parameter of interest, in the MAP approach the posterior results are used to obtain the predictive distribution of the parameter in the new trial. This predictive distribution of the parameter in the new trial is then used as an informative prior for the analysis of the observed data in the new trial. The predictive distribution accounts for both the between‐trial heterogeneity and the uncertainty in the estimation of the population mean parameter.^2^, ^3^, ^4^, ^5^

The MAP approach has been implemented for different types of data, including log‐odds for binary outcomes,^2^ mean count for overdispersed count data in negative binomial models,^3^ and effect of active control for normal outcomes.^4^ However in all these applications, only a single parameter, typically the intercept or rate, was assumed to vary across trials. This assumption may not be realistic in more complex statistical models. No implementation of the MAP approach is available in a multivariate setting, where multiple parameters can vary across studies simultaneously.

In randomized controlled trials (RCTs) with a pretest–posttest design, researchers measure an endpoint at baseline and typically the same endpoint as an outcome at follow‐up. Here we focus on continuous endpoints. The analysis of covariance (ANCOVA) model is a linear regression model that adjusts for the baseline effect to estimate the treatment effect on the health outcome at follow‐up.^6^ In ANCOVA models, both the estimates for the intercept and the baseline effect affect the treatment effect estimate. Therefore it is sensible to allow for variation in both parameters when implementing the MAP approach in ANCOVA models, which leads to an extension of the MAP approach to a multivariate setting.

In previous implementations, the MAP approach often considered summary statistics instead of individual participant data (IPD), that is, the data recorded for each participant in a study. Although the MAP approach could be implemented based on the reported summary statistics in some settings, for example, binomial models, it is unlikely that publications report the required sufficient statistics for ANCOVA models.^7^ The summary statistics from publications may also be unreliable due to low quality reporting and publication bias.^8^, ^9^, ^10^, ^11^, ^12^ Furthermore, historical studies may differ from the current study in many important aspects (including study design, research group, inclusion criteria), but it is difficult to rule out highly heterogeneous historical trials under scenarios where the IPD are not available. To avoid the aforementioned problems we assume that IPD are available for all historical studies.

The purpose of this study is to extend the MAP approach to a multivariate setting, which allows for multiple study‐specific random effects, in ANCOVA models with IPD data available. The major challenge of the extension is in the estimation of the between‐study covariance matrix based on a limited number of historical trials.^13^ This makes it more favorable to implement the multivariate MAP in a Bayesian framework, so that external evidence and historical information may be incorporated in the model in a flexible manner.

The motivating data in this study were RCTs on Alzheimer's disease from the UC San Diego Alzheimer's Disease Cooperative Study (ADCS).^14^ The trials were conducted by the same organization with similar inclusion criteria, which justifies the incorporation of information from preceding trials in a new trial.

The content of this paper is organized as follows. Section 2 provides the basic information of the motivating ADCS data. Section 3 introduces the methodology for the MAP approach and describes the extension of this approach to ANCOVA models. Section 4 evaluates the performance of the proposed extension with a simulation study. Section 5 illustrates the implementation of the proposed MAP approach for ANCOVA models using the motivating ADCS data, and Section 6 summarizes and discusses the main findings.

## 2 MOTIVATING DATA

Alzheimer's disease, a neurodegenerative disorder, is among the most important health problems in aging populations worldwide and the most common cause of dementia.^15^ The Alzheimer's Association has estimated that the number of Alzheimer's disease patients worldwide will rise to more than 100 million worldwide by 2050.^16^ This could impose a heavy burden on healthcare systems due to its long duration and costly healthcare service. So far, no drugs are available for treating behavioral and psychiatric symptoms that may develop in the moderate and severe stages of Alzheimer's disease.^17^ Furthermore, clinical trials for Alzheimer's disease are difficult to conduct due to (1) the slow pace of recruiting sufficient numbers of participants, and (2) the relatively long time needed to observe whether an investigational treatment affects disease progression.^18^ Given this context, historical borrowing methods may prove useful for the analysis of RCTs on Alzheimer's disease.

To investigate potential treatments for the symptoms, UC San Diego set up a research network named Alzheimer's Disease Cooperative Study (ADCS). This network has conducted a number of RCTs that evaluate compounds that may benefit the well‐being of Alzheimer's disease patients. In this study, data from six RCTs, namely ADC‐011, ADC‐015, ADC‐016, ADC‐022, ADC‐027, and ADC‐037, were retrieved from the ADCS to illustrate the implementation of the MAP approach.^19^, ^20^, ^21^, ^22^, ^23^, ^24^ Among the six trials considered, the most recent trial, that is, ADC‐037, is regarded as the new trial, and the remaining five trials are treated as historical trials. All six trials were similar with respect to the treatment for control arm patients, but each trial evaluated a different compound for the treatment arm. The baseline characteristics of the trials are shown in Table 1.

**TABLE 1:** Baseline characteristics of the candidate studies.

Study	ADC‐011	ADC‐015	ADC‐016	ADC‐022	ADC‐027	ADC‐037
Number of subjects (*T*/*C*)	351 (240/111)	406 (204/202)	409 (240/169)	313 (153/160)	402 (238/164)	119 (64/55)
Study period	1999–2000	2002–2007	2003–2006	2005–2009	2007–2009	2012–2014
Study duration (month)	14	20	18	26	18	13
Baseline age (mean (*SD*))	73.9 (7.7)	74.6 (9.3)	76.9 (8.0)	76.2 (7.9)	75.9 (8.5)	71.3 (8.1)
Sex (% of female)	53.0	59.4	56.0	58.8	52.2	57.1
Years of education (mean (*SD*))	14.0 (3.3)	14.3 (3.2)	13.9 (3.1)	13.8 (3.3)	14.3 (2.8)	15.1 (3.0)
Baseline MMSE (mean (*SD*))	20.8 (3.7)	20.3 (4.7)	20.9 (3.5)	16.9 (2.9)	20.7 (3.6)	20.4 (4.4)
Baseline ADAS‐cog (mean (*SD*))	24.3 (9.6)	24.6 (10.1)	23.0 (9.0)	30.2 (9.3)	24.2 (8.9)	24.5 (9.5)

*Abbreviations*: *T*, treatment arm; *C*, control arm; *SD*, standard deviation.

In total, there were 806 subjects in the control arms of the above historical trials, and the baseline characteristics for the Alzheimer's disease patients across trials were fairly comparable. The cognitive score, ADAS‐cog, of the patients was measured at baseline and 12 months after the treatment. Assuming that the change of ADAS‐cog in 1 year is of interest, an ANCOVA model that adjusts for the baseline ADAS‐cog score could be fitted to estimate the effect of the randomized treatment. The trials are similar in terms of study population, year in which the study conducted, type of study design, definition of the outcome, and quality of study execution and management. Pocock's comparability criteria,^25^ which have been considered a prerequisite for the use of historical borrowing methods, are mostly satisfied for these trials. We thus conclude that the analysis of the new trial (ADC‐037) with the MAP approach is feasible for the ADCS data sets.

## 3 METHOD

In this section, the methodology to implement the MAP approach in the design and analysis of pretest–posttest trial data is elaborated, including the ANCOVA model, the basics of the MAP approach, and the methodological details of the proposed MAP approach in ANCOVA models.

### 3.1 The ANCOVA model

In a trial with a pretest–posttest design, where the response is measured at baseline and at follow‐up, an ANCOVA model can be used to estimate the treatment effect by adjusting for the baseline outcome. The model is given by

(1) $$y_{\mathrm{Fi}}=\beta_{0}+\beta_{1}y_{\mathrm{Bi}}+\lambda {\mathrm{trt}}_{i}+\epsilon_{i},$$

where $y_{\mathrm{Fi}}$ denotes the response of the *i*th subject at the follow‐up, $\beta_{0}$ is the intercept, $\beta_{1}$ is the effect of pre‐treatment measurement on the post‐treatment measurement, that is, the baseline effect, $y_{\mathrm{Bi}}$ denotes the baseline measurement for the *i*th subject, λ is the treatment effect, trt_*i*_ is the treatment assignment for the *i*th subject (trt_*i*_ 0 for the control arm, and 1 for the treatment arm), and $\epsilon_{i}∼N\left(0,\sigma_{\epsilon }^{2}\right)$ is the random error.

Note that in some cases, the treatment effect may be influenced by the baseline score. In the ANCOVA model, this effect modification could be represented by an interaction term between the baseline score and the treatment assignment. If necessary, the MAP approach for the ANCOVA model can be extended to include this interaction term. But the MAP prior will stay the same, because the interaction term plays no role when only the historical controls are considered. In this study, we chose not to include the interaction in that it may complicate the inference for the treatment effect and the interpretation of the results.

### 3.2 Basics of the MAP approach

The MAP approach is based on the assumption of exchangeable parameters in the new trial and historical trials, in which the effects in different studies are different but from the same normal distribution.^2^

Suppose that we only account for variation between trials with respect to the intercept $\beta_{0}$ of the ANCOVA model. The parameter estimates of the intercept within each historical trial should be approximately normally distributed, so that

(2) $${\hat{\beta }}_{0j}|\beta_{0j}∼N\left(\beta_{0j},\sigma_{0j}^{2}\right),$$

where $\beta_{0j}$ and ${\hat{\beta }}_{0j}$ are the study‐specific intercept for the *j*th historical trial and its estimate, and $\sigma_{0j}^{2}$ is the variance of ${\hat{\beta }}_{0j}$. Then if there are J historical control trials with study‐specific intercepts $\beta_{01},\ldots ,\beta_{0J}$ and one new trial with intercept $\beta_{0}^{*}$, the MAP approach assumes that these parameters are distributed as

(3) $$\beta_{01},\ldots ,\beta_{0J},\beta_{0}^{*}|\mu_{0}∼N\left(\mu_{0},\tau_{0}^{2}\right),$$

where $\mu_{0}$ is the grand mean, and $\tau_{0}^{2}$ is the between‐study variance of the study‐specific means.

For known $\tau_{0}^{2},$ the grand mean $\mu_{0}$ is distributed as

(4) $$\mu_{0}|{\hat{\beta }}_{01},\ldots ,{\hat{\beta }}_{0J},\tau_{0}∼N\left(\frac{\sum w_{0j}{\hat{\beta }}_{0j}}{\sum w_{0j}},\frac{1}{\sum w_{0j}}\right),$$

where $w_{0j}=\frac{1}{\sigma_{0j}^{2}+\tau_{0}^{2}}$ is the inverse‐variance weight for the *j*th historical trial. For meta‐analysis, inference for $\mu_{0}$ is of primary interest, whereas the MAP approach aims to derive an informative prior for $\beta_{0}^{*}$ in the new trial, which is given by

(5) $$\beta_{0}^{*}|{\hat{\beta }}_{01},\ldots ,{\hat{\beta }}_{0J},\tau_{0}∼N\left(\frac{\sum w_{0j}{\hat{\beta }}_{0j}}{\sum w_{0j}},\frac{1}{\sum w_{0j}}+\tau_{0}^{2}\right).$$

The variance of the above MAP prior is the combination of the variance of the grand mean and the between‐study variance. However, the between‐study variance (or heterogeneity) is not known in practice. The estimation of the between‐study heterogeneity can be based on either frequentist (e.g., DerSimonian‐Laird's method, ML/REML) or Bayesian methods. However, it cannot be inferred with great precision if the number of historical trials is limited.^26^

In the above paragraphs, the MAP approach is described based on the two‐stage meta‐analytic approach, where the study‐specific estimates are derived from each study in advance, and the MAP prior is then obtained based on the study‐specific estimates. This was done to illustrate the effects of the within‐study variation and the between‐study variation for the parameter of interest in the MAP approach.^27^ If the IPD are available, a one‐stage approach that simultaneously analyzes the historical control data can be used to derive the MAP prior.

In addition to the proposal of the MAP prior in the design phase of a new trial, the meta‐analytic methodology can be directly implemented in the analysis of new trial data, which is called the meta‐analytic‐combined (MAC) approach.^28^

### 3.3 MAP for ANCOVA models

In ANCOVA models, the estimation of the intercept and the baseline effect affects the estimate of the treatment effect. In a MAP approach applied to an ANCOVA model, it would be natural to allow for variation in the intercept across studies, to account for between‐study differences in the mean outcome at follow‐up. However, it is unlikely that the baseline effects would be consistent across studies while the intercepts vary, because typically the same health outcome is measured at baseline and at follow‐up in a pretest–posttest trial. Therefore we account for possible variation in both the intercept and the baseline effect across studies in a MAP prior for an ANCOVA model. Previous implementations of the MAP approach only focused on variation in a single parameter, and our approach thus consists of an extension of the MAP approach to a bivariate setting.

In this study, the implementation of the MAP approach will be based on the availability of IPD, using a one‐stage meta‐analytic approach with data from multiple trials analyzed in a single hierarchical model.^29^ To explain the MAP approach for the ANCOVA model, a meta‐analysis with only historical controls serves as the starting point. In the one‐stage random effects meta‐analysis, data from different studies are analyzed simultaneously. Note that only historical controls are involved, that is, only the intercept and the baseline effect are considered in this setting. A one‐stage meta‐analysis, assuming IPD are available, can be fitted as follows

(6) $$y_{\mathrm{Fij}}=\beta_{0j}+\beta_{1j}y_{\mathrm{Bij}}+\epsilon_{\mathrm{ij}},$$

where the subscript i denotes the patient number within a trial and the subscript j denotes the trial number, the intercept $\beta_{0j}$ and the baseline effect $\beta_{1j}$ are study‐specific effects with

(7) $$\left(\begin{array}{c} \beta_{0j}\\ \beta_{1j} \end{array}\right)∼\mathrm{MVN}\left(\left(\begin{array}{c} \mu_{0}\\ \mu_{1} \end{array}\right),\sum_{\beta }\right),\sum_{\beta }=\left(\begin{array}{cc} \tau_{0}^{2} & \rho_{\tau }\tau_{0}\tau_{1}\\ \rho_{\tau }\tau_{0}\tau_{1} & \tau_{1}^{2} \end{array}\right).$$

In the above bivariate normal distribution, $\mu_{0}$ and $\mu_{1}$ are the overall mean of the intercept and the baseline effect respectively, $\tau_{0}^{2}$ and $\tau_{1}^{2}$ are the between‐study variances, and $\rho_{\tau }$ is the correlation between the intercept and the baseline effect across studies.^13^, ^30^ The error term $\epsilon_{\mathrm{ij}}∼N\left(0,\sigma_{\epsilon j}^{2}\right)$, where $\sigma_{\mathrm{\varepsilon j}}^{2}$ is the trial‐specific error variance for the *j*th trial.

Meta‐analysis is usually conducted to synthesize the information on treatment effects from multiple studies. Note that in the meta‐analysis for the aforementioned ANCOVA model, only between‐study variation in the parameters for the control arms is modeled, and no treatment effect can be estimated from only the controls. The aim of the MAP approach is to improve the estimation of the treatment effect by combining an informative prior based on a meta‐analytic model for the historical controls with the data of the current, randomized trial.

Because we assume IPD are available, we implement the MAP approach for ANCOVA using a one‐stage approach. The one‐stage MAP model is actually a linear mixed model with study‐specific intercepts and baseline effects. The predictive distribution for the intercept and the baseline effect in the new trial $\left(\beta_{0}^{*},\beta_{1}^{*}\right),$ given the grand means $\mu_{0}$ and $\mu_{1}$ and the covariance matrix $\sum_{\beta },$ is

(8) $$\left(\begin{array}{c} \beta_{0}^{*}\\ \beta_{1}^{*} \end{array}\right)∼\mathrm{MVN}\left(\left(\begin{array}{c} \mu_{0}\\ \mu_{1} \end{array}\right),\sum_{\beta }\right),$$

where the grand means and the covariance matrix are treated as random variables in a Bayesian context, and uncertainty of their estimation is considered in the estimation of $\beta_{0}^{*}$ and $\beta_{1}^{*}.$

Different model specifications are possible with the MAP approach for ANCOVA models in that we can have different specifications for the between‐study variation in the intercept and the baseline effect. In the main version of our proposed approach, we allow for different variances of the intercept and the baseline effect and correlation between the two (i.e., an unstructured covariance matrix $\sum_{\beta }).$ Alternatively we could assume the two parameters to be independent, that is, $\rho_{\tau }=0$ or an intercept‐only univariate MAP, in which a common baseline effect is assumed for different studies. The latter approach was suggested by Neuenschwander et al. in case study‐specific covariates are available.^2^ However, this model ignores the clustering of patients and heterogeneous adjustment factors across studies, which may lead to misleading effect estimates and conclusions.^29^, ^31^ For example, results of a meta‐analysis for logistic regression models can be downwardly biased when ignoring the clustering.^31^ Another model specification can be obtained by specifying an intercept‐only univariate MAP, in which there are separate baseline effects for the different studies.

### 3.4 Estimation

In principle the parameters of the MAP approach for ANCOVA models could be estimated using either maximum likelihood or in a Bayesian way. However when there is a limited number of historical trials, which makes the estimation of parameters for the between‐study heterogeneity difficult, the Bayesian approach seems more appropriate than the frequentist approach, because the Bayesian approach can incorporate the available historical information in a flexible manner, with suitably chosen priors.^32^ It may even be impossible for a frequentist approach to estimate a 2×2 covariance matrix with just a few historical trials.^13^, ^33^ In this study, the Bayesian approach will thus be used.

For the regression coefficients, normal priors with fairly large variances can be used for the overall means of the regression coefficients $\left(\mu_{0},\mu_{1}\right).$ Note that the baseline effect $\beta_{1}=r_{y_{F},y_{B}}\frac{s_{y_{F}}}{s_{y_{B}}}$, where $r_{y_{F},y_{B}}$ is the correlation between $y_{B}$ and $y_{F},$ $s_{y_{F}}$ is the standard deviation of $y_{F},$ and $s_{y_{B}}$ is the standard deviation of $y_{B}.$ Therefore, only $\beta_{0}$ and not $\beta_{1}$ is affected by the scaling (e.g., the unit of measurement) of the outcome. The following priors were chosen for $\mu_{0}$ and $\mu_{1}:$ $\mu_{0}∼N\left(0,10^{4}\right)$ and $\mu_{1}∼N\left(0,10^{2}\right)$. An inverse Gamma prior was specified for the error variance in the *j*th trial $\left(\sigma_{\mathrm{\varepsilon j}}^{2}\right),$ that is, $\sigma_{\mathrm{\varepsilon j}}^{2}∼\mathrm{Inv}-\text{Gamma}\left(10^{-3},10^{-3}\right)$.

The above priors are commonly used noninformative priors for fitting Bayesian linear regression models, and the posteriors are not sensitive to deviations from the above priors because the available data is far more informative than the priors. Unlike the specification of priors for the above parameters, specifying the prior for the between‐study covariance matrix $\left(\sum_{\beta }\right)$ is an essential part of the MAP approach. The prior for the covariance matrix $\sum_{\beta }$ may considerably influence the posterior results, especially when the number of historical trials is limited. Based on previous literature on multivariate meta‐analysis, an inverse Wishart prior for the between‐study covariance matrix is not recommended due to its strong constraints on the variance parameters,^13^, ^34^, ^35^ which may lead to erroneous estimates for the between‐study variations and potential type I/II errors. As a result, a separation strategy that gives separate priors for the between‐study standard deviations and the correlation coefficient will be used in this study for more robust estimation and more flexibility in incorporating prior information.

For the prior of the correlation coefficient $\rho_{\tau }$, not much prior knowledge is available, and uniform priors have often been selected for between‐study correlation coefficients in multivariate meta‐analysis.^30^ However Burke et al. have found that the routine use of a Uniform (‑1,1) prior should be avoided, as it is not necessarily vague, and that sensible prior distributions are preferred, for example by restricting values to be positive or negative as indicated by prior knowledge.^30^ Based on the structure of the ANCOVA model and the results obtained for the ADCS data (shown later), we expect the value of $\rho_{\tau }$ to be negative, so that the intercept and the baseline effect are negatively correlated. Therefore we specify a Uniform(−1,0) prior for $\rho_{\tau }.$

Unlike the specification of priors for the aforementioned parameters, it is more challenging to specify a sensible prior for the between‐study heterogeneity, that is, $\tau_{0}^{2}$ and $\tau_{1}^{2}.$ Especially if the number of historical trials is low, the results may be sensitive to the prior.^13^ For the estimation of $\tau_{0}^{2}$ and $\tau_{1}^{2},$ the traditional inverse Gamma prior is best avoided.^30^ Based on previous studies, a prior that assigns most of its probability mass over a range of plausible between‐study standard deviations (*τ*_0_ and *τ*_1_) was recommended.^3^, ^36^ We follow recommendations of previous authors to use a half‐normal prior that assigns 5% probability to large between‐study heterogeneity, which implies no historical information is incorporated.^2^, ^37^ For regression coefficients, the between‐study heterogeneity is often considered large when the between‐study standard deviation equals the within‐study standard deviation. The latter is calculated by multiplying the study‐specific standard error of the regression coefficient by the square root of the number of subjects.^2^, ^4^ For comparable historical trials, the within‐study standard deviations are often similar. We therefore choose the scale parameter of the half‐normal prior for the between‐study standard deviation such that the 95th percentile corresponds with the maximum of the derived within‐study standard deviations. This prior specification assigns 5% prior probability to the situation that the historical studies are not relevant, and should also give a reasonable distribution over low, medium and high heterogeneity. In the following simulation study and motivating data analysis, different half‐normal priors will be used for *τ*_0_ and *τ*_1_. In addition, sensitivity analysis for the shape of the prior, for instance, exponential distributions or uniform distributions with 5% probability to large between‐study heterogeneity, could be considered.

### 3.5 The effective sample size of the MAP prior

Although the MAP prior for the parameters in the new trial is of most interest in the design phase, it does not have a direct interpretation in the design phase. In other words, it is important to know the amount of information introduced via this informative prior, which could be quantified in terms of the number of patients, that is, the prior effective sample size (ESS).^38^ This concept is a useful quantification of the amount of information from historical controls in Bayesian models.

For the MAP prior, it is not straightforward to quantify the information contained in a prior distribution as a number of patients. To solve this problem, Neuenschwander et al. proposed a variance‐ratio method that assumes the information is proportional to the precision.^2^ The method is valid when the prior distribution is approximately normal which applies in our setting, and this was verified in the analysis of the ADCS data.

The calculation of the prior ESS using the variance‐ratio method is based on a comparison of the variance of $\beta_{0}$ or $\beta_{1}$ between the analysis of the historical controls when where is no between‐study heterogeneity (i.e., *τ* = 0) and the MAP prior in which a positive τ is estimated from the data. We denote the variance of the prior distribution of $\beta_{i}$ (*i* = 0,1) as $V_{\beta_{i},0},$ when the value τ is fixed to 0, and it is denoted as $V_{\beta_{i},\tau }$ when τ is estimated from the data. If there is no between‐study heterogeneity, the prior ESS is the total number of historical controls, that is, *N*. The prior ESS for $\beta_{i}$ according to the variance‐ratio method is then given by $n_{\beta_{i}}^{*}=\frac{V_{\beta_{i},0}}{V_{\beta_{i},\tau }}N.$

## 4 SIMULATION

In this section, a simulation study is presented to evaluate the performance of the MAP approach for ANCOVA models and to compare this method with alternative approaches, such as ignoring the historical controls and pooling the historical data with the new trial data.

### 4.1 Simulation setting

The simulated historical data sets and the new trial data set were generated based on the following ANCOVA model with study‐specific random effects

(9) $$y_{\mathrm{Fij}}=\beta_{0j}+\beta_{1j}y_{\mathrm{Bij}}+\lambda {\mathrm{trt}}_{\mathrm{ij}}+\epsilon_{\mathrm{ij}},$$

where $\beta_{0j}$ denotes the intercept in the *j*th study (*j* = 1⋯*J*) for historical trials, *j* = *J* + 1 for the new trial), and $\beta_{1j}$ is the baseline effect in the *j*th study. The study‐specific effects have a multivariate normal distribution with mean $\left(\mu_{0},\mu_{1}\right)$ and the covariance matrix $\sum_{\beta }=\left(\begin{array}{cc} \tau_{0}^{2} & \rho_{\tau }\tau_{0}\tau_{1}\\ \rho_{\tau }\tau_{0}\tau_{1} & \tau_{1}^{2} \end{array}\right)$.

The parameters in the above model were specified based on the data of the ADC‐037 trial. Therefore, $\mu_{0}=1.06,\;\mu_{1}=1.16,\;y_{\mathrm{Bij}}∼N\left(24.53,9.47^{2}\right),\;\text{and}\;\epsilon_{\mathrm{ij}}∼N\left(0,6.79^{2}\right).$ To evaluate frequentist characteristics, scenarios with and without treatment effect were considered, with λ=0 to simulate no treatment effecgt, and λ=−3 to simulate a treatment, which was based on the minimal clinically relevant change of ADAS‐cog in 1 year.^39^ The treatment effect λ was not included in the data‐generating model for historical control data.

Different scenarios for the between‐study heterogeneity were considered in the simulations. Given that the baseline mean and standard deviation of the ADAS‐cog were the same across trials in the data generating process, within‐trial standard deviations for $\beta_{0}$ and $\beta_{1}$ were approximately constant across trials, which were approximately 20 and 0.72, respectively. Different levels of between‐study heterogeneity were quantified based on the ratio of the between‐trial standard deviation to the within‐trial standard deviation (*τ*/*σ*). Therefore data were generated for five scenarios for the between‐study heterogeneity, with $\tau_{0}$ and $\tau_{1}$ in the data generation set to the values in Table 2. The between‐study correlation $\rho_{\tau }$ was −0.9 in all scenarios.

**TABLE 2:** Different levels of the between‐study heterogeneity in the simulation study.

Level	*τ*/*σ*	*τ*_0_	*τ*_1_
No	0	0	0
Small	1/8	2.5	0.09
Moderate	1/4	5	0.18
Substantial	1/2	10	0.36
Large	1	20	0.72

Sixty subjects per arm for three or five historical control arms were simulated. There were a total of 5×2×2=20 scenarios in the simulation study.

Six models were included and evaluated in the simulation study, including (1) no borrowing (ignoring the historical controls), (2) the multivariate MAP with correlated parameters (MMAP+COR), (3) the multivariate MAP with independent parameters (MMAP+IND), (4) the univariate MAP with a common baseline effect (UMAP+COM), (5) the univariate MAP with separate baseline effects (UMAP+SEP), and (6) pooling (combining the historical controls with the current trial without adjustment). To explicitly illustrate the structures of the aforementioned MAP approaches, distributions of the intercept and the baseline effect in the new trial for these approaches are shown in Table 3.

**TABLE 3:** Distributions of the intercept and the baseline effect in the new trial for the MAP approaches.

Approach	Distribution
MMAP+COR	$\left(\begin{array}{c} \beta_{0}^{*}\\ \beta_{1}^{*} \end{array}\right)∼\mathrm{MVN}\left(\left(\begin{array}{c} \mu_{0}\\ \mu_{1} \end{array}\right),\left(\begin{array}{cc} \tau_{0}^{2} & \rho_{\tau }\tau_{0}\tau_{1}\\ \rho_{\tau }\tau_{0}\tau_{1} & \tau_{1}^{2} \end{array}\right)\right)$
MMAP+IND	$\left(\begin{array}{c} \beta_{0}^{*}\\ \beta_{1}^{*} \end{array}\right)∼\mathrm{MVN}\left(\left(\begin{array}{c} \mu_{0}\\ \mu_{1} \end{array}\right),\left(\begin{array}{cc} \tau_{0}^{2} & 0\\ 0 & \tau_{1}^{2} \end{array}\right)\right)$
UMAP+COM	$\beta_{0}^{*}∼N\left(\mu_{0},\tau_{0}^{2}\right)$, shared $\beta_{1}$ across studies
UMAP+SEP	$\beta_{0}^{*}∼N\left(\mu_{0},\tau_{0}^{2}\right)$, different $\beta_{1}$ across studies

The priors for the model parameters were as follows. The prior for the intercept and the treatment effect was N(0,10^4^), while the prior for the baseline effect was N(0,10^2^). The prior for the error variances was Inv‐Gamma(10^‑3^,10^‑3^), which were allowed to vary across studies. The prior for the between‐study covariance matrix $\sum_{\beta }$ was specified with a separation strategy. The priors for the between‐study standard deviations of the intercept and the baseline effect were half‐normal with scale parameters 10 and 0.36, so that the 95th percentile corresponds with the τ values for large heterogeneity in Table 2. The prior for the between‐study correlation was Uniform(‑1, 0).

In each simulation scenario, 1000 simulated data sets were generated and analyzed with the above six models respectively. Using Markov chain Monte Carlo (MCMC) sampling in JAGS, a total of 16,000 samples were drawn for each simulated data set after a burn‐in phase of 1000 iterations in each of the four chains. All methods achieved good convergence in the simulation.

To compare the different methods, bias, standard deviation (*SD*) and root mean squared error (RMSE) of the treatment effect were calculated as well as the type I error rate and statistical power. In scenarios without treatment effect, the type I error occurred if the 95% credible interval of the treatment effect estimate did not include zero. In scenarios with treatment effect, the type II error was defined if the 95% credible interval of the treatment effect included zero. To assess the amount of historical information obtained from the above methods in the design phase, the prior ESS for the intercept and the baseline effect was calculated using the variance‐ratio method.

### 4.2 Simulation results

The prior ESS and its 95% credible interval for the intercept and the baseline effect in simulation scenarios without treatment effect are presented in Table 4. The prior ESS in scenarios with treatment effect were similar, and therefore presented in Table S1 of Supporting Information.

**TABLE 4:** The prior ESS (95% credible interval) for the intercept and the baseline effect for the MAP approaches in scenarios without treatment effect.

			Between‐study heterogeneity				
Parameter	*J*	Method	No	Small	Moderate	Substantial	Large
Intercept	3	MMAP+COR	14 (7, 23)	11 (4, 19)	8 (3, 17)	5 (2, 16)	5 (2, 14)
		MMAP+IND	16 (8, 24)	12 (4, 22)	8 (2, 19)	5 (2, 17)	4 (2, 13)
		UMAP+COM	25 (12, 41)	19 (8, 38)	14 (5, 33)	10 (4, 31)	8 (4, 24)
		UMAP+SEP	10 (5, 15)	9 (3, 15)	7 (2, 15)	5 (2, 15)	4 (2, 13)
	5	MMAP+COR	61 (25, 102)	34 (9, 77)	15 (4, 46)	6 (3, 17)	4 (2, 10)
		MMAP+IND	90 (39, 145)	52 (12, 115)	20 (4, 71)	6 (2, 21)	4 (2, 10)
		UMAP+COM	120 (48, 188)	74 (23, 160)	36 (11, 118)	16 (7, 49)	10 (6, 23)
		UMAP+SEP	31 (11, 55)	21 (6, 48)	11 (3, 39)	5 (2, 16)	4 (2, 10)
Baseline effect	3	MMAP+COR	14 (7, 22)	12 (5, 19)	8 (3, 17)	6 (2, 17)	6 (2, 21)
		MMAP+IND	16 (8, 24)	13 (4, 22)	9 (3, 19)	6 (2, 17)	5 (2, 19)
	5	MMAP+COR	61 (23, 102)	36 (10, 79)	16 (5, 51)	7 (3, 23)	5 (3, 17)
		MMAP+IND	92 (38, 147)	54 (12, 115)	22 (4, 72)	7 (2, 28)	5 (2, 15)

For the intercept, the UMAP+COM led to the largest prior ESS among all the MAP approaches in all simulation scenarios, which was followed by the MMAP+IND approach, the true underlying MMAP+COR approach, and the UMAP+SEP approach. Regarding the baseline effect, the MMAP+IND yielded larger prior ESS for the MMAP+COR. The results indicated that a misspecified MAP model did not necessarily lead to a low prior ESS, and operating characteristics of the inference for the parameter of interest may be more appropriate in the evaluation of MAP approaches.

All methods yielded unbiased estimates of the treatment effect as expected, the detailed results are shown in Table S2 of Supporting Information. Standard deviations of the treatment effect for different methods in simulated scenarios without treatment effect are shown in Table 5. Pooling the data yielded the highest precision except when the between‐study heterogeneity was large. Among the historical borrowing methods, the UMAP+COM yielded the lowest standard deviation with no to moderate between‐study heterogeneity, followed by the MMAP+COR, the MMAP+IND, and the UMAP+SEP. With substantial to large between‐study heterogeneity, the underlying true MMAP+COR model yielded the highest precision among all the MAP approaches. In case of large between‐study heterogeneity, the UMAP+COM method, which assumes no variation in the baseline effect, gave a less precise estimate of the treatment effect than even no borrowing. It is also noteworthy that the MMAP+IND had higher standard deviations compared to the MMAP+COR although it had larger prior ESS for $\beta_{0}$ and $\beta_{1}$, which implied that larger prior ESS of the parameters did not necessarily lead to a more precise treatment effect estimate. Standard deviations in simulated scenarios with treatment effect are shown in Table S3.

**TABLE 5:** The standard deviation of the treatment effect estimate for different methods in simulated scenarios without treatment effect.

		Between‐study heterogeneity				
*J*	Method	No	Small	Moderate	Substantial	Large
3	No borrowing	1.254	1.256	1.256	1.252	1.250
	MMAP+COR	1.172	1.198	1.227	1.241	1.247
	MMAP+IND	1.185	1.209	1.235	1.247	1.249
	UMAP+COM	1.136	1.173	1.216	1.246	1.286
	UMAP+SEP	1.231	1.238	1.246	1.247	1.250
	Pooling	0.984	0.993	1.022	1.122	1.454
5	No borrowing	1.255	1.253	1.254	1.256	1.250
	MMAP+COR	1.125	1.166	1.213	1.243	1.246
	MMAP+IND	1.137	1.176	1.227	1.252	1.249
	UMAP+COM	1.089	1.140	1.204	1.252	1.284
	UMAP+SEP	1.225	1.231	1.243	1.253	1.249
	Pooling	0.950	0.961	0.996	1.121	1.516

Root mean squared errors of the treatment effect are presented in Table 6. Among the historical borrowing methods, the UMAP+COM approach had the lowest RMSE with no to moderate between‐study heterogeneity among all the MAP approaches, while the MMAP+COR yielded the lowest RMSE when the between‐study heterogeneity was substantial or large. The UMAP+COM yielded a RMSE larger than that of no borrowing with large between‐study heterogeneity. As expected, pooling gave lower RMSEs than the other methods in the scenario without heterogeneity, and strongly increased RMSEs with substantial or large heterogeneity. Root mean squared errors in simulated scenarios with treatment effect are shown in Table S4.

**TABLE 6:** The root mean squared error of the treatment effect estimate for different methods in simulated scenarios without treatment effect.

		Between‐study heterogeneity				
*J*	Method	No	Small	Moderate	Substantial	Large
3	No borrowing	1.257	1.227	1.226	1.275	1.260
	MMAP+COR	1.128	1.142	1.184	1.261	1.254
	MMAP+IND	1.146	1.160	1.200	1.266	1.260
	UMAP+COM	1.083	1.116	1.176	1.268	1.288
	UMAP+SEP	1.224	1.207	1.215	1.269	1.259
	Pooling	0.986	1.097	1.375	2.159	3.772
5	No borrowing	1.196	1.308	1.259	1.245	1.266
	MMAP+COR	0.989	1.178	1.198	1.223	1.259
	MMAP+IND	1.004	1.192	1.221	1.242	1.264
	UMAP+COM	0.951	1.153	1.181	1.234	1.304
	UMAP+SEP	1.148	1.278	1.244	1.242	1.263
	Pooling	0.893	1.078	1.247	1.824	3.435

To compare the methods in terms of the hypothesis testing of the treatment effect, the type I error rates and the statistical powers are shown in Table 7 and Table 8, respectively.

**TABLE 7:** The type I error rate of the treatment effect estimate for different methods.

		Between‐study heterogeneity				
J	Method	No	Small	Moderate	Substantial	Large
3	No borrowing	5.5	5.0	4.3	6.1	6.0
	MMAP+COR	5.0	4.0	4.3	5.3	5.9
	MMAP+IND	4.5	4.3	4.4	5.7	5.9
	UMAP+COM	4.7	4.2	4.7	5.7	5.6
	UMAP+SEP	4.3	5.1	4.4	5.4	5.8
	Pooling	5.1	7.4	14.4	31.1	45.8
5	No borrowing	4.0	6.0	6.0	6.2	4.8
	MMAP+COR	2.4	5.1	5.5	5.7	5.0
	MMAP+IND	2.3	5.3	6.0	5.9	4.7
	UMAP+COM	2.3	4.7	4.9	6.3	5.0
	UMAP+SEP	3.5	5.7	5.8	6.0	5.1
	Pooling	4.4	8.6	10.9	22.5	39.2

**TABLE 8:** The statistical power of the treatment effect estimate for different methods.

		Between‐study heterogeneity				
*J*	Method	No	Small	Moderate	Substantial	Large
3	No borrowing	65.6	66.4	67.9	69.1	68.5
	MMAP+COR	71.4	70.8	71.6	69.1	68.5
	MMAP+IND	70.7	69.3	70.8	68.7	68.6
	UMAP+COM	74.0	73.6	71.0	69.4	65.8
	UMAP+SEP	67.0	66.8	69.4	69.3	68.8
	Pooling	84.5	83.2	78.4	65.0	61.6
5	No borrowing	66.8	65.2	68.6	66.5	67.1
	MMAP+COR	79.2	72.9	71.9	67.6	66.6
	MMAP+IND	78.4	72.2	70.8	66.9	66.7
	UMAP+COM	82.6	74.9	72.8	67.6	64.2
	UMAP+SEP	69.3	66.8	69.2	66.4	67.1
	Pooling	89.9	86.4	81.7	67.4	53.7

As can be seen from Table 7, all MAP approaches controlled the type I error rate. However, the pooling approach inflated the type I error rate even with small between‐study heterogeneity.

Based on Table 8, pooling is the best way to analyze the data without between‐study heterogeneity because it yielded the highest power and controlled the type I error rate in the meantime. Under no to small between‐study heterogeneity, the UMAP+COM approach yielded the highest power among all the MAP approaches, followed by the MMAP+COR. The two approaches were comparable in terms of performance with moderate to substantial between‐study heterogeneity. The power of the UMAP+COM approach was even lower than that without borrowing when the between‐study heterogeneity was large.

To summarize, the proposed MMAP+COR approach performed properly in terms of the operating characteristics in all the simulated scenarios, whereas other MAP models were inferior to the MMAP+COR approach in specific scenarios, for example, the MMAP+IND and UMAP+SEP approaches with no to substantial between‐study heterogeneity and UMAP+COM with substantial to large between‐study heterogeneity. However it should be noted that these other models were ‘misspecified’, in the sense that the data were generated according to the MMAP+COR model.

## 5 MOTIVATING DATA ANALYSIS

In this section, we illustrate the implementation of the extended MAP approach, that is, MMAP+COR, in the new pretest–posttest trial (ADC‐037 in the ADCS data) on Resveratrol. Resveratrol is a phytoalexin produced by several plants that acts against pathogens, such as bacteria and fungi,^40^ there has been evidence that it decreases aging‐related cognitive decline and pathology based on Alzheimer's disease animal models.^41^, ^42^ Hence, it is of interest to conduct an RCT to investigate its effect on the cognitive function of patients with mild to moderate Alzheimer's disease.^24^

### 5.1 Design

**FIGURE 1:**
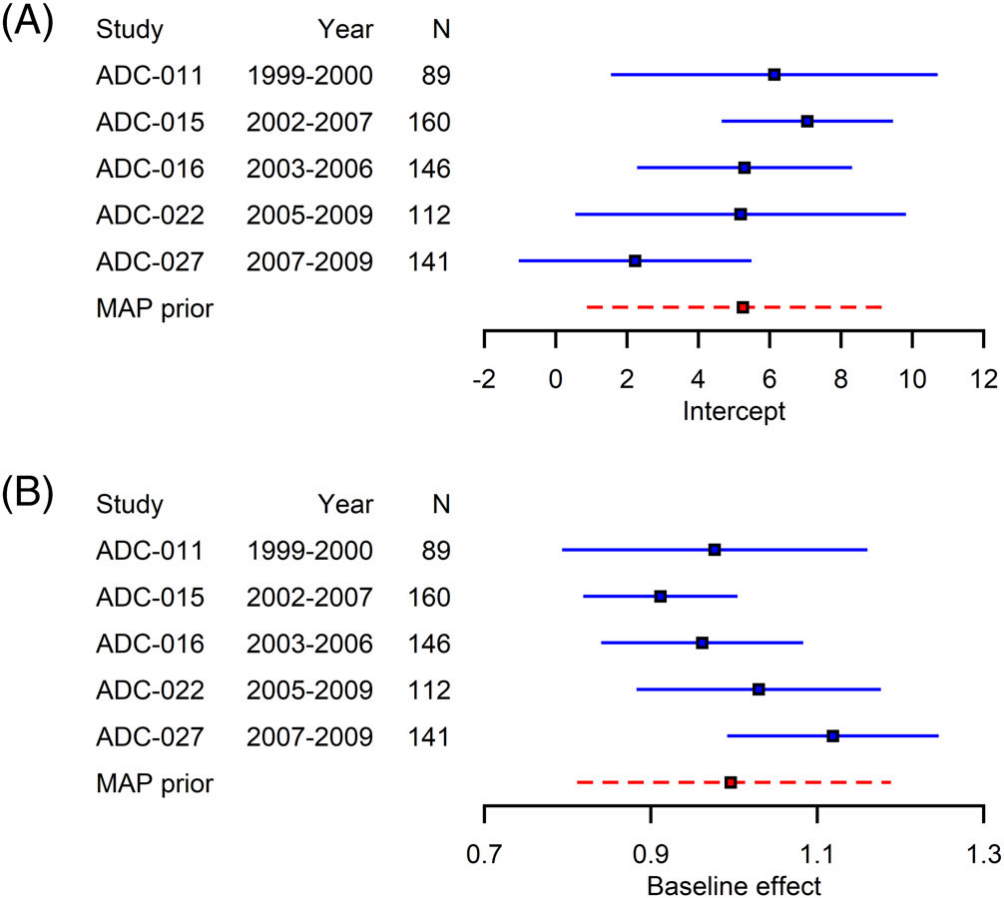
Estimates (95% credible intervals) of (a) the intercept and (b) the baseline effect in five historical controls and their corresponding MAP priors (red, dashed). [Colour figure can be viewed at wileyonlinelibrary.com]

Before using the MAP approach to construct predictive distributions for the intercept and the baseline effect, we first derived trial‐specific estimates and the corresponding standard errors of these parameters using ANCOVA models for each historical trial separately. In these ANCOVA models, the subjects with missing follow‐up outcomes were excluded, which left a total of 648 historical controls. The derived standard errors were multiplied by the square roots of the numbers of observations in order to specify the half‐normal priors for the between‐study standard deviations. The study‐specific estimates (95% credible interval) of the intercept and the baseline effect from the abovementioned ANCOVA models are shown in the forest plots in Figure 1.

As can be seen from the forest plots, the intercept (*β*_0_) and the baseline effect (*β*_1_) had a negative correlation which provided evidence in favor of the implementation of a multivariate MAP approach that accounts for their correlation. Based on the results, the between‐study standard deviation representing large between‐study heterogeneity was approximately 20 for *β*_0_, and about 1 for *β*_1_. Therefore, the prior for the between‐study standard deviation of the intercept (*τ*_0_) was half‐normal with scale parameter 10, and the prior for the between‐study standard deviation of the baseline effect (*τ*_1_) was half‐normal with scale parameter 1/2. The priors for other model parameters were specified as in Section 3.4. The analysis was done with MCMC using JAGS, a total of $2\times 10^{5}$ samples were drawn with four chains, and the method achieved good convergence.

**FIGURE 2:**
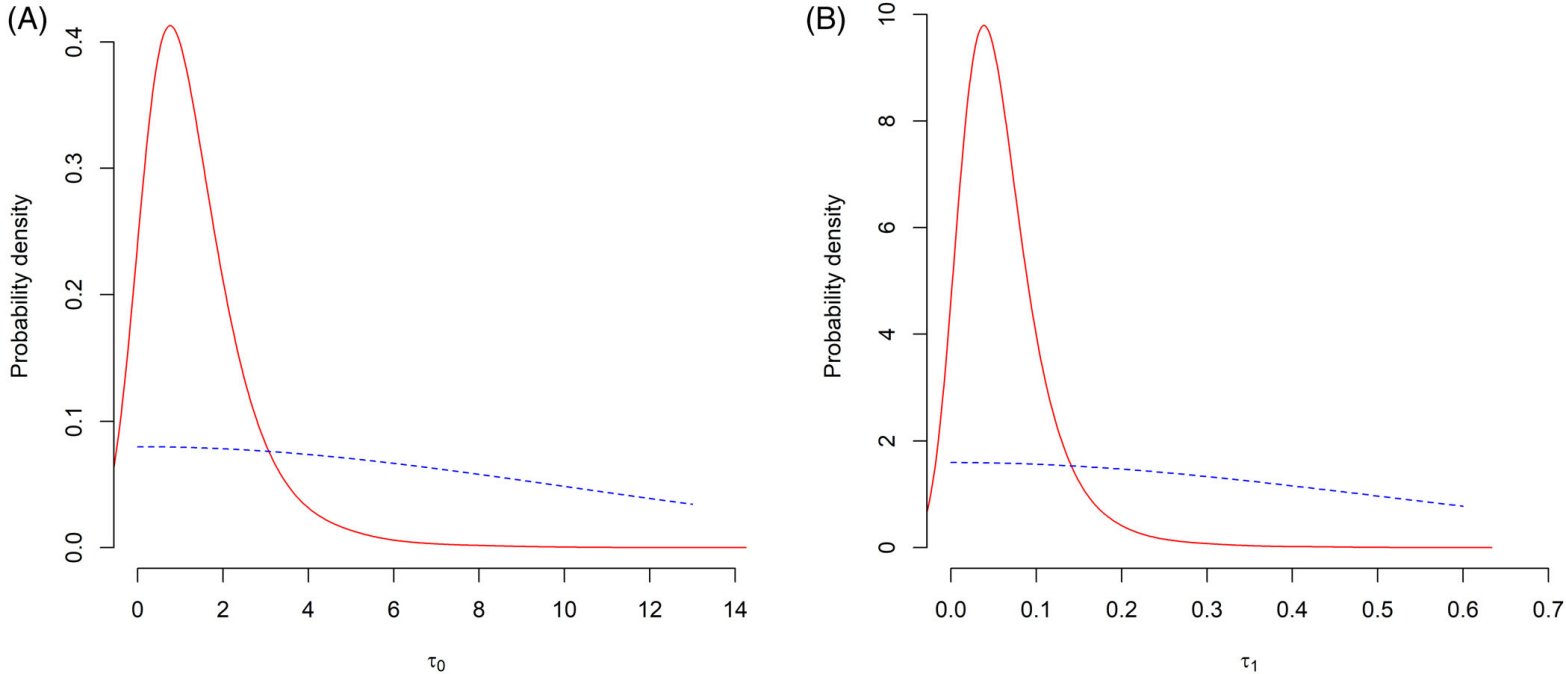
Priors (blue, dashed) and posteriors (red, solid) of the between‐study heterogeneity for (a) the intercept and (b) the baseline effect. [Colour figure can be viewed at wileyonlinelibrary.com]

The priors and posteriors of the between‐study standard deviations for $\beta_{0}$ and $\beta_{1}$ are shown in Figure 2. The posteriors assigned quite low probability to large between‐study heterogeneity. The posterior modes of $\tau_{0}$ and $\tau_{1}$ were 1.38 and 0.05, respectively, and these values correspond with small between‐study heterogeneity. These results imply that the multivariate MAP methods borrowed the historical information to a non‐negligible extent.

**FIGURE 3:**
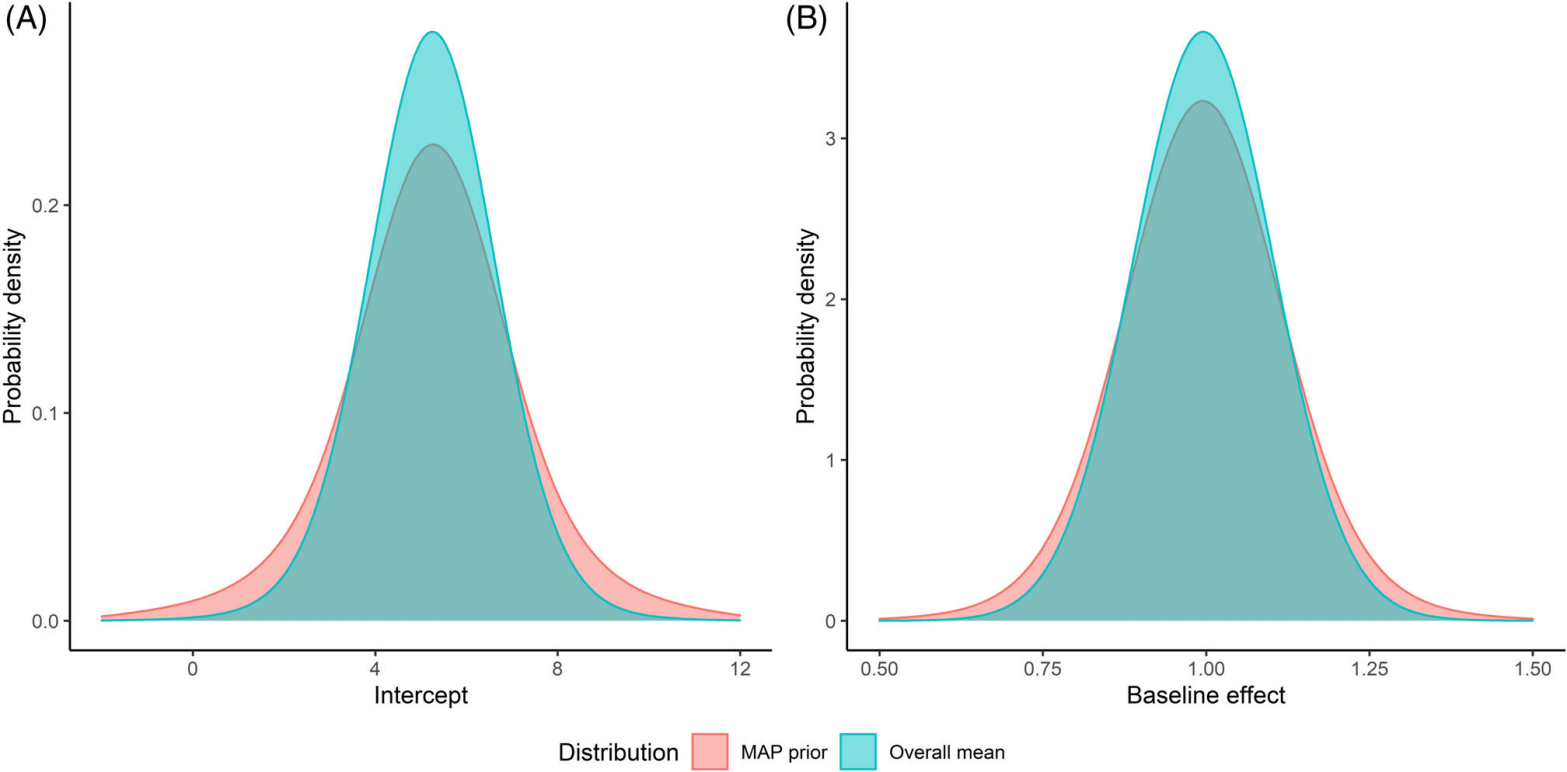
Posteriors of the overall means and MAP priors for (a) the intercept and (b) the baseline effect based on five historical control arms. [Colour figure can be viewed at wileyonlinelibrary.com]

The posteriors for $\mu_{0}$ and $\mu_{1}$ derived from the meta‐analysis of historical controls and the MAP priors for $\beta_{0}^{*}$ and $\beta_{1}^{*}$ are visualized in Figure 3. For each parameter, means of the posterior and the MAP prior were the same, while the variance of the MAP prior was derived by combining the variance of the posterior and the between‐trial variance of the parameter. The priors were much more informative than weakly informative normal priors with large variations specified for the overall means.

The estimates for $\beta_{0}$ and $\beta_{1}$ by pooling the historical controls were 5.00 (*SD*: 0.73) and 1.01 (*SD*: 0.03), respectively. The MAP prior for $\beta_{0}$ was approximately normal with a mean of 5.25, a standard deviation of 2.11, and a prior ESS of 79, while the MAP prior for $\beta_{1}$ had a mean of 1.00, a standard deviation of 0.09, and a prior ESS of 58. The prior ESS for $\beta_{0}$ and $\beta_{1}$ were different, which suggests that the historical controls provided more information for the intercept than for the baseline effect.

### 5.2 Analysis

In this study, we already had the data for the new trial, that is, ADC‐037, so we also illustrated the implementation of the MAP approach in the analysis of the new trial. This analysis was also done with MCMC using the same settings as in Section 5.1. The estimates of the model parameters for (1) no borrowing, (2) MMAP+COR, and (3) pooling are presented in Table 9.

**TABLE 9:** Estimates of model parameters based on No borrowing, MMAP+COR approach and pooling.

Method	Parameter	Mean	*SD*	95% credible interval
No borrowing	$\beta_{0}$	1.06	1.91	−2.69 to 4.81
	$\beta_{1}$	1.16	0.07	1.02 to 1.30
	λ	−1.10	1.35	−3.74 to 1.55
MMAP+COR	$\beta_{0}$	3.57	1.52	0.09 to 6.04
	$\beta_{1}$	1.07	0.06	0.97 to 1.20
	λ	−1.27	1.18	−3.60 to 1.04
Pooling	$\beta_{0}$	4.44	0.69	3.10 to 5.79
	$\beta_{1}$	1.03	0.03	0.98 to 1.08
	λ	−1.15	0.93	−2.97 to 0.67

By incorporating the MAP priors in the analysis, the standard deviations for the intercept, the baseline effect, and the treatment effect were reduced by 20.4%, 14.3%, and 12.6%, which indicated that the MAP approach can effectively improve the precision of the parameter of interest with compatible historical control data.

**FIGURE 4:**
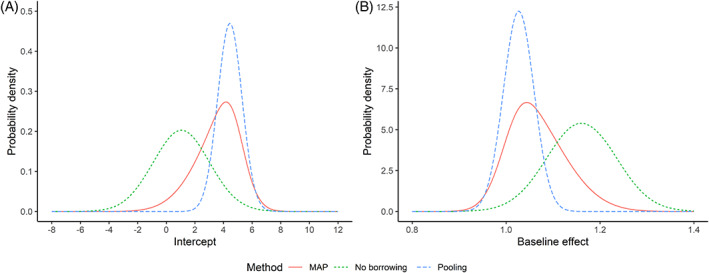
Posteriors of (a) the intercept and (b) the baseline effect based on no borrowing, MMAP+COR, and pooling. [Colour figure can be viewed at wileyonlinelibrary.com]

The posteriors of the intercept and the baseline effect in the new trial based on the above three approaches are visualized in Figure 4. These figures show that the inclusion of historical data considerably changes the estimates of the intercept and the baseline effect.

**FIGURE 5:**
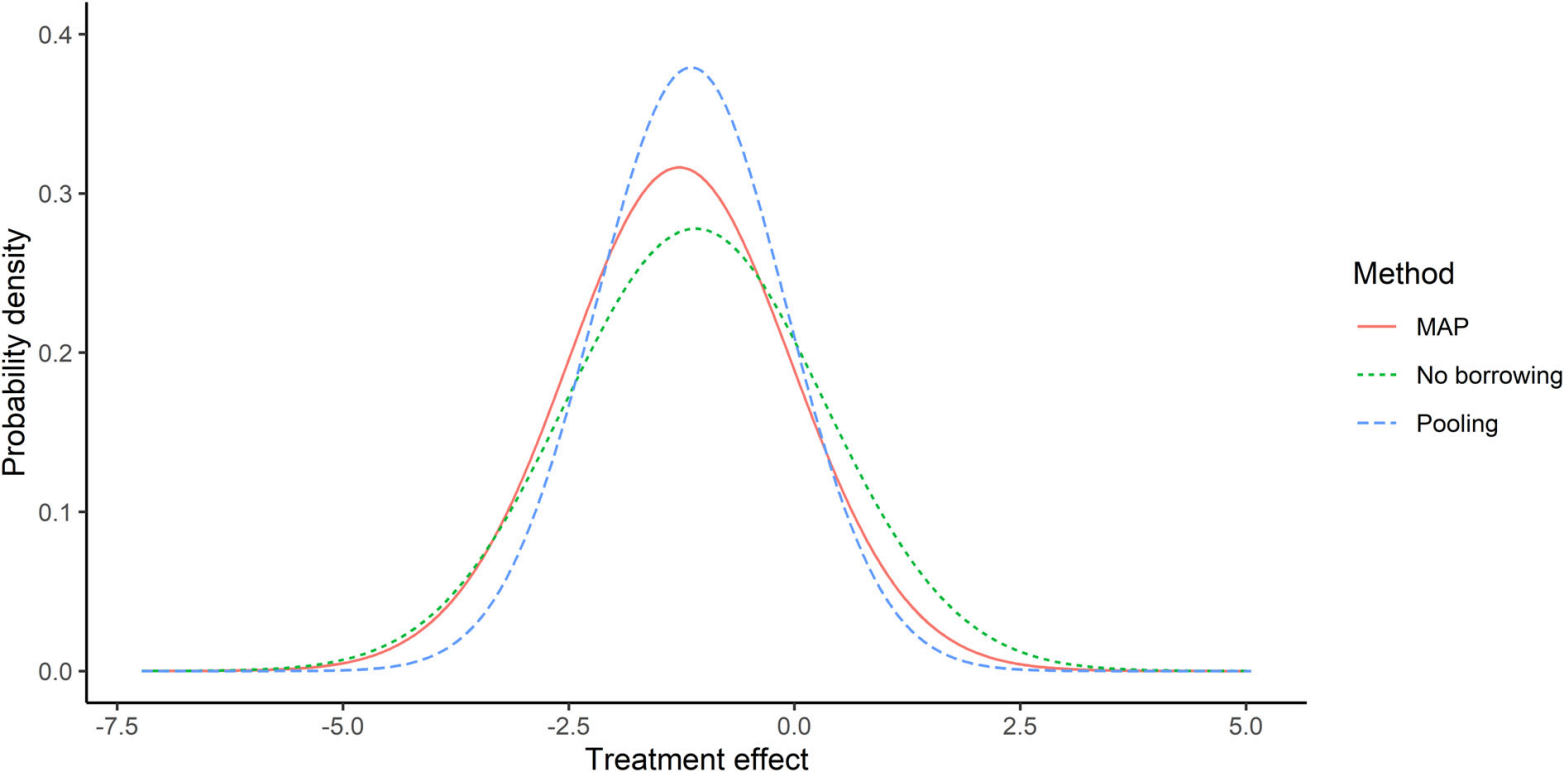
Posteriors of the treatment effect based on no borrowing, MMAP+COR, and pooling. [Colour figure can be viewed at wileyonlinelibrary.com]

The posteriors of the treatment effect are visualized in Figure 5. The estimates of the treatment effect were similar, but the precision of the treatment effect estimate in the MAP approach was higher than that without borrowing.

A sensitivity analysis was conducted to assess the robustness of the proposed MAP approach to different priors for the between‐study heterogeneity in accordance with Section 3.4. The results showed that the proposed MAP approach was robust to the choice of exponential or uniform prior with the same tail probability. Furthermore, different MAP approaches were also included in the sensitivity analysis, the inference for the treatment effect in the MMAP+IND approach was similar to that in the MMAP+COR approach while the results of the univariate MAP (UMAP+COM and UMAP+SEP) deviated from the results of the MMAP+COR. Detailed results of the sensitivity analysis and JAGS scripts for the motivating data analysis are presented in the second and third sections  Supporting Information, respectively.

## 6 DISCUSSION

In this study, we extended the MAP approach to a multivariate version by incorporating the information of multiple parameters from historical trials in the analysis of a new clinical trial. Based on the simulation results, the proposed MAP approach which accounts for the between‐study heterogeneity and the correlation between multiple parameters could improve the estimation of the parameter of interest (i.e., the treatment effect) with greater precision, increased power, while keeping type I error rates at acceptable levels. The motivating data analysis also showed that the proposed MAP approach led to increased precision for the treatment effect estimate.

Regarding the inference for the treatment effect, the UMAP+COM yielded the highest precision, lowest RMSE, and highest power among all the MAP approaches if there was no to small between‐study heterogeneity. In terms of these results the UMAP+COM approach was followed by the MMAP+COR approach. When the between‐study heterogeneity was higher, the MMAP+COR had the best inference for the treatment effect, whereas the UMAP+COM even led to power loss compared to no borrowing. The explanation is that the UMAP+COM may have a more precise treatment effect estimate by ignoring the clustering of different studies when the between‐study heterogeneity is relatively small, but it may harm the inference of the target parameter if the between‐study heterogeneity becomes larger. Besides, ignoring the clustering across studies requires a rather strong assumption, which is widely criticized in the context of meta‐analysis.^29^ Based on the results, we prefer the proposed MMAP+COR approach in the analysis of pretest–posttest trials using ANCOVA models over the other proposed MAP methods. This method seems the safest choice across different levels of between‐study heterogeneity.

To our knowledge the MAP approach has so far only been implemented for a single parameter. For regression models, Di Scala et al. implemented the MAP approach to derive the informative prior for the effect of an active control versus placebo in a hierarchical model with a patient‐level random intercept for a cross‐over study. Other model parameters were assumed to differ across studies, and precision of the treatment effect was improved.^4^ The results in our study have shown that the statistical inference for the parameter of interest could be further improved if information of multiple parameters in the model is incorporated, which justifies the extension in this study.

The main challenge of extending the MAP approach to a multivariate setting lies in the estimation of the between‐study heterogeneity for the included model parameters, especially if the number of historical trials is limited. In this regard, the Bayesian approach is preferred over the frequentist approach because the former incorporates prior knowledge of the between‐study heterogeneity in a more flexible way,^33^, ^43^ and it allows the user to specify a sensible prior that gives weight to both lower and higher possible differences between trials. The half‐normal prior for the between‐study standard deviation with 5% probability assigned to large between‐study heterogeneity seems a reasonable choice since it is weakly informative and can cover a wide range of realistic values.^30^ Also the simulation results show that this prior is skeptical enough to avoid inflation of the type I error rate. In both the simulation study and the ADCS data, the estimation of three parameters in the covariance matrix $\sum_{\beta }$ was not a problem even with a relatively low number of historical studies.

The results suggest that the bivariate MAP approach might be extended to the context of more than two parameters, where the separation strategy can still be utilized when eliciting the prior for the between‐study covariance matrix. This may for example make sense in linear regression models where not just the intercept but also the regression coefficients could conceivably vary across trials. To account for between‐study differences in more than two model parameters, reparameterization, such as Cholesky decomposition, is required for the correlation matrix to ensure that it is positive semidefinite. However the estimation of a large covariance matrix may be troublesome with a limited number of historical controls. A structured covariance matrix could then be used to ease the burden of estimation.^35^

In this study, informative priors for multiple parameters were derived based on the proposed MAP approach, and multiple corresponding prior ESS values were calculated based on the MAP priors. Based on the simulation, the true underlying model does not lead to the highest ESS, which implies that the improved precision of the priors does not necessarily yield improved inference for the treatment effect. Further research is needed to assess the value of the prior ESS for the design and sample size calculation of the new trial.

There are some limitations in the study. First, the simulation settings were specified according to the ADCS data in this study, and model parameters in other realistic cases may differ from those used in the simulation study, which may lead to slightly different results with the proposed MAP approach. However, main findings in this study could still hold if the historical controls are comparable based on the comparability criteria. Second, the study mainly focused on the analysis of the new trial using the MAP approach but did not cover much about its role in the new trial design. In previous studies, priors of the model parameters were used to inform the sample size calculation of new trials in the Bayesian framework.^27^, ^44^ Therefore, further studies could be conducted to assess the usefulness of the MAP approach in the Bayesian sample size determination of new trials by synthesizing the historical evidence.

In conclusion, the MAP approach can be extended to a bivariate setting even with a limited number of historical trials (e.g., 3). The most promising results were obtained by simultaneously considering historical information from multiple parameters and their correlation in the statistical inference of ANCOVA models.

## CONFLICT OF INTEREST

The authors declare no conflicts of interest.

## AUTHOR CONTRIBUTIONS

**Hongchao Qi**: conceptualization, methodology, software, formal analysis, and writing original draft. **Dimitris Rizopoulos**: conceptualization, methodology, review and editing of the manuscript, and supervision. **Joost van Rosmalen**: conceptualization, methodology, review and editing of the manuscript, and supervision.

## Supporting information


**Appendix S1** Supporting Information

## Data Availability

The R and JAGS scripts for the simulation study can be found on GitHub at https://github.com/QiHongchao/MAP_ANCOVA_publication. The ADCS data could be requested from the ADCS Data and Sample Sharing Committee.
