# Supplementary material for: Incorporating historical control information in ANCOVA models using the meta‐analytic‐predictive approach
Source: Res Synth Methods. 2022 Apr 26;13(6):681–96. doi: 10.1002/jrsm.1561 (PMC9790567; doi:10.1002/jrsm.1561)
Supplement: Supplementary file 1 — Appendix S1 Supporting Information [file JRSM-13-681-s001.pdf]

Supplementary document for “Incorporating historical control information in ANCOVA models using the meta-analytic-predictive approach”

Hongchao Qi*[1, 2], Dimitris Rizopoulos[1, 2], and Joost van Rosmalen[1, 2]

[1]Department of Biostatistics, Erasmus University Medical Center

[2]Department of Epidemiology, Erasmus University Medical Center

In this supplementary document, additional results of the simulation study are presented in Section 1. Section 2 provides results of the sensitivity analysis for the motivating example, and Section 3 presents JAGS scripts for the motivating data analysis.

1 Additional results of the simulation study

In this section, the additional results of the simulation, including prior ESS, bias, standard deviation, and root mean squared error are presented in Table S1 to Table S4.

*Correspondence: Hongchao Qi, Doctor Molewaterplein 40, 3015 GD Rotterdam, the Netherlands. Email: h.qi@erasmusmc.nl. Data used in the study were obtained from the University of California, San Diego Alzheimer's Disease Cooperative Study, https://www.adcs.org/.

Table S1: The prior ESS of the intercept and the baseline effect for the MAP approaches in scenarios with treatment effect

Parameter	J	Method	Between-study heterogeneity				
			No	Small	Moderate	Substantial	Large
Intercept	3	MMAP+COR	14 (7, 22)	11 (4, 20)	8 (3, 17)	5 (2, 16)	5 (2, 19)
		MMAP+IND	16 (7, 25)	13 (4, 22)	8 (2, 19)	5 (2, 16)	5 (2, 17)
		UMAP+COM	25 (11, 42)	20 (7, 38)	14 (5, 34)	9 (4, 32)	8 (4, 25)
		UMAP+SEP	10 (5, 15)	9 (3, 15)	6 (2, 15)	5 (2, 15)	4 (2, 16)
	5	MMAP+COR	61 (24, 102)	36 (9, 82)	14 (4, 45)	6 (3, 19)	4 (2, 11)
		MMAP+IND	91 (37, 147)	53 (9, 121)	19 (4, 62)	6 (2, 26)	4 (2, 10)
		UMAP+COM	120 (46, 186)	74 (22, 159)	35 (11, 117)	16 (7, 47)	11 (6, 28)
		UMAP+SEP	32 (11, 55)	22 (6, 50)	11 (3, 38)	5 (2, 18)	4 (2, 10)
Baseline effect	3	MMAP+COR	15 (7, 22)	12 (5, 20)	8 (3, 18)	6 (2, 18)	5 (2, 17)
		MMAP+IND	16 (8, 25)	13 (4, 23)	9 (3, 19)	6 (2, 18)	5 (2, 16)
	5	MMAP+COR	61 (23, 104)	37 (9, 82)	16 (5, 50)	7 (3, 26)	5 (2, 12)
		MMAP+IND	91 (41, 145)	55 (9, 119)	22 (4, 76)	7 (2, 30)	4 (2, 12)

Table S2: The bias of the treatment effect estimate for different methods in the simulation

J	Trt	Method	Between-study heterogeneity				
			No	Small	Moderate	Substantial	Large
3	No	No borrowing	0.015	−0.005	−0.012	0.035	0.010
		MMAP+COR	0.000	−0.009	−0.007	0.033	0.008
		MMAP+IND	0.003	−0.011	−0.003	0.031	0.006
		UMAP+COM	−0.009	−0.007	−0.009	0.034	0.019
		UMAP+SEP	0.012	−0.011	−0.006	0.034	0.007
		Pooling	−0.041	−0.014	0.015	−0.014	0.136
	Yes	No borrowing	0.074	−0.001	−0.036	−0.065	−0.001
		MMAP+COR	0.067	0.002	−0.035	−0.058	0.002
		MMAP+IND	0.070	0.004	−0.036	−0.056	−0.001
		UMAP+COM	0.065	0.004	−0.032	−0.055	0.015
		UMAP+SEP	0.076	0.005	−0.035	−0.060	−0.002
		Pooling	0.044	−0.001	−0.059	−0.053	−0.137
5	No	No borrowing	−0.072	−0.021	0.015	−0.051	0.031
		MMAP+COR	−0.054	−0.012	0.007	−0.050	0.029
		MMAP+IND	−0.056	−0.010	0.009	−0.051	0.029
		UMAP+COM	−0.049	−0.008	0.012	−0.044	0.025
		UMAP+SEP	−0.071	−0.019	0.012	−0.050	0.030
		Pooling	−0.037	−0.014	0.007	−0.058	0.012
	Yes	No borrowing	−0.035	0.025	−0.058	0.017	−0.003
		MMAP+COR	−0.056	0.014	−0.061	0.017	0.003
		MMAP+IND	−0.057	0.007	−0.063	0.013	0.001
		UMAP+COM	−0.062	0.014	−0.057	0.014	0.005
		UMAP+SEP	−0.042	0.011	−0.062	0.014	0.001
		Pooling	−0.069	0.004	−0.049	0.039	−0.066

Table S3: The standard deviation of the treatment effect estimate for different methods in simulated scenarios with treatment effect

J	Method	Between-study heterogeneity				
		No	Small	Moderate	Substantial	Large
	No borrowing	1.255	1.251	1.249	1.251	1.252
	MMAP+COR	1.174	1.194	1.220	1.241	1.248
	MMAP+IND	1.186	1.204	1.228	1.247	1.251
	UMAP+COM	1.138	1.168	1.207	1.248	1.285
	UMAP+SEP	1.233	1.234	1.239	1.248	1.251
	Pooling	0.984	0.992	1.021	1.123	1.440
	No borrowing	1.251	1.254	1.251	1.252	1.252
	MMAP+COR	1.121	1.168	1.211	1.238	1.248
	MMAP+IND	1.132	1.178	1.224	1.247	1.250
	UMAP+COM	1.083	1.142	1.202	1.247	1.283
	UMAP+SEP	1.220	1.232	1.240	1.248	1.250
	Pooling	0.949	0.962	0.995	1.121	1.517

Table S4: The root mean squared error of the treatment effect estimate for different methods in simulated scenarios with treatment effect

J	Method	Between-study heterogeneity				
		No	Small	Moderate	Substantial	Large
	No borrowing	1.264	1.250	1.219	1.208	1.235
	MMAP+COR	1.136	1.171	1.184	1.191	1.230
	MMAP+IND	1.157	1.187	1.198	1.200	1.236
	UMAP+COM	1.095	1.146	1.177	1.198	1.270
	UMAP+SEP	1.234	1.233	1.212	1.203	1.236
	Pooling	0.982	1.121	1.353	2.171	3.968
	No borrowing	1.215	1.252	1.211	1.247	1.318
	MMAP+COR	1.018	1.120	1.143	1.222	1.308
	MMAP+IND	1.031	1.139	1.166	1.236	1.312
	UMAP+COM	0.980	1.100	1.131	1.235	1.335
	UMAP+SEP	1.166	1.221	1.197	1.238	1.313
	Pooling	0.924	1.040	1.219	1.846	3.282

2 Results of the sensitivity analysis for the motivating data analysis

In the sensitivity analysis, the proposed MAP approach was implemented with different priors for the between-study standard deviations of β_0 and β_1 to assess the method's robustness to different priors of between-study heterogeneity, and the results with different model specifications were also derived.

2.1 Different priors for the between-study standard deviations

In this part of the sensitivity analysis, exponential and uniform priors that assign 5% probability for large between-study heterogeneity were considered. Namely, the exponential priors for τ_0 and τ_1 were Exp(0.15) and Exp(3), and the uniform priors for τ_0 and τ_1 were Uniform(0, 21.05) and Uniform(0, 1.05), respectively. The above priors along with the half-normal priors used in the main analysis are visualized in Figure S1.

Despite the same probability assigned to large between-study heterogeneity, the exponential prior assigns more probability to small between-study heterogeneity than the half-normal prior, while the uniform prior provides less probability to small between-study heterogeneity. Hence, the exponential prior express more confidence for a small between-study heterogeneity and the uniform prior is the least informative among the three priors.

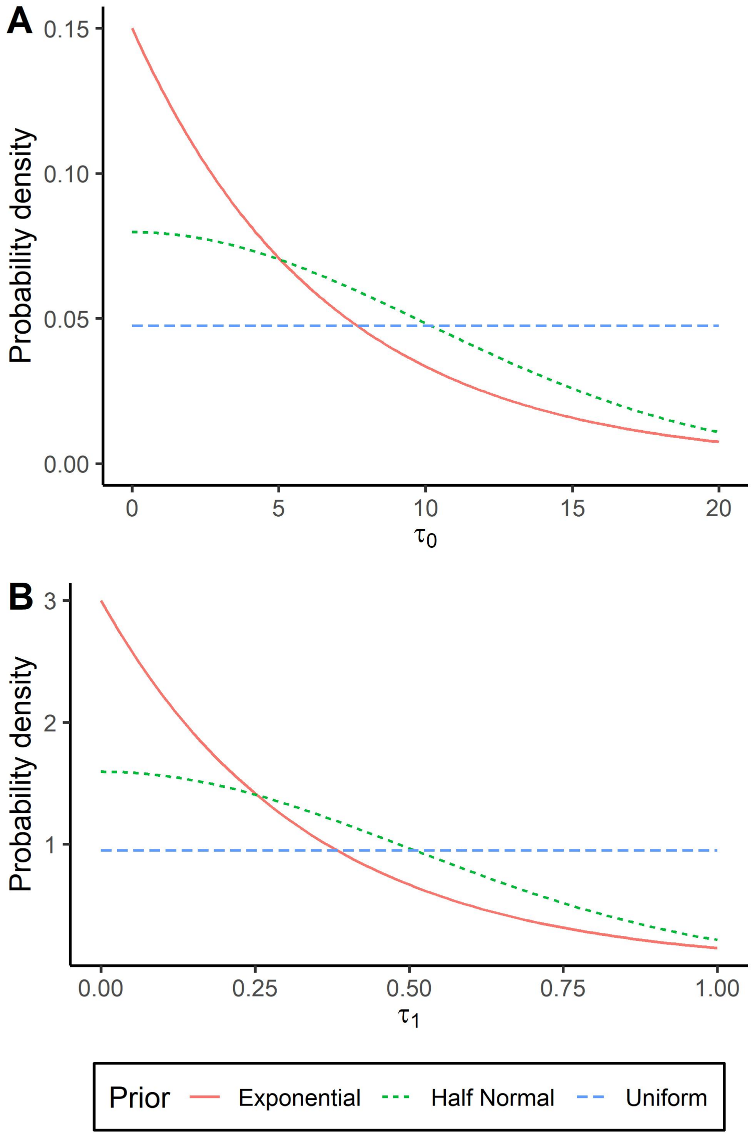

Figure S1: Different priors for between-study standard deviations of (A) the intercept (τ_0) and (B) the baseline effect (τ_1)

The prior ESS for the intercept was 108, and the prior ESS for the baseline effect was 75 with the exponential priors. While the prior ESS for the intercept and the baseline effect were 72 and 48 respectively based on the uniform priors. The prior ESS for the two parameters based on half-normal priors (79 and 58) were between those with uniform priors and exponential priors. The results were reasonable in that the exponential prior is more informative while the uniform prior is less informative than the half-normal prior. The MAP priors and the prior ESS are shown in Table S5.

Table S5: The MAP priors and prior ESS for the intercept and the baseline effect with exponential and uniform priors for the between-study standard deviations

Prior	Parameter	Mean	SD	95% credible interval	ESS
Exponential	β_0	5.27	1.80	1.58 to 8.90	108
	β_1	0.99	0.08	0.84 to 1.16	75
Uniform	β_0	5.26	2.21	0.74 to 9.67	72
	β_1	1.00	0.10	0.80 to 1.20	48

The parameter estimates based on the exponential and uniform priors are presented in Table S6. The treatment effect estimate with the exponential priors was -1.27 (SD: 1.16), and the treatment effect estimate based on the uniform priors was -1.28 (SD: 1.18). The estimates were similar to that based on the half-normal priors, which indicated the inference of the parameter of interest was robust to differently shaped priors (but same tail probability) for the between-study standard deviations.

Table S6: The estimates of model parameters with exponential and uniform priors for the between-study standard deviations

Prior	Parameter	Mean	SD	95% credible interval
Exponential	β_0	3.74	1.43	0.41 to 6.03
	β_1	1.06	0.06	0.97 to 1.19
	λ	-1.27	1.16	-3.56 to 1.02
Unifrom	β_0	3.55	1.53	0.08 to 6.00
	β_1	1.07	0.06	0.97 to 1.20
	λ	-1.28	1.18	-3.60 to 1.02

2.2 Different MAP approaches

In the simulation study, there were three other MAP approaches, namely MMAP+IND, UMAP+COM, and UMAP+SEP considered. The results of the abovementioned models are also presented in Table S7 and Table S8.

The prior ESS of the intercept and the baseline effect with the MMAP+IND were larger than those in the MMAP+COR. The UMAP+COM yielded the largest prior ESS for the intercept, while the UMAP+SEP led to the smallest. The results were in line

with those in the simulation study.

Table S7: The MAP priors and prior ESS for the intercept and the baseline effect based on different MAP approaches

Prior	Parameter	Mean	SD	95% credible interval	ESS
MMAP+IND	β_0	5.39	1.62	2.28 to 8.51	133
	β_1	0.99	0.07	0.86 to 1.13	102
UMAP+COM	β_0	5.32	1.30	2.95 to 7.93	207
UMAP+SEP	β_0	5.27	3.04	−1.01 to 11.42	38

The inference for the treatment effect in the MMAP+IND was similar to that in the MMAP+COR, while the treatment effect estimates of the UMAP+COM and the UMAP+SEP were more deviated from that in the MMAP+COR.

Table S8: The estimates of model parameters based on different MAP approaches

Model	Parameter	Mean	SD	95% credible interval
MMAP+IND	β_0	4.48	1.03	2.10 to 6.29
	β_1	1.03	0.04	0.96 to 1.13
	λ	−1.30	1.19	−3.67 to 1.02
UMAP+COM	β_0	4.65	0.82	3.01 to 6.28
	β_1	1.02	0.03	0.96 to 1.07
	λ	−1.06	1.07	−3.16 to 1.03
UMAP+SEP	β_0	2.80	1.78	−0.96 to 5.83
	β_1	1.11	0.07	0.98 to 1.24
	λ	−1.46	1.36	−4.12 to 1.21

3 JAGS scripts for the motivating data analysis

In this section, JAGS scripts for ‘No borrowing”, “Pooling”, and ‘MMAP+COR” approaches used in the motivating data analysis of Section 5 are presented. “No borrowing” and “Pooling” approaches share the same JAGS scripts, while the “MMAP+COR” approach has its unique JAGS scripts.

3.1 JAGS scripts for "No borrowing" and "Pooling" approaches

In this subsection, JAG scripts for "No borrowing" and "Pooling" approaches are presented. To implement the two approaches, a single data set is needed (either the new trial data only or the pooled data without study identifier).

```
model {
##Priors
##Prior for the intercept and baseline effect, bivariate normal with low precision
beta ~ dmnorm(mean_beta, prec_beta)
##Treatment effect
lambda ~ dnorm(0, 1e-4)
##Error precision
tau ~ dgamma(1e-3, 1e-3)
##Likelihood
for (i in 1:N) {
response[i] ~ dnorm(mu[i], tau)
mu[i] <- fixef[i,] %*% c(beta, lambda)
}
}
```

3.2 JAGS scripts for the "MMAP+COR" approach

In this subsection, JAGS scripts for the "MMAP+COR" approach in both design and analysis of the new trial are presented. The scripts for the design phase which only include the historical controls are presented below.

```
model {
##Historical likelihood
##Each of H historical studies
for (i in 1:H){
##Intercept and baseline effect in ith historical study
beta_historical[i,1:num_fixef] ~ dmnorm(beta, Omega_beta)
##Error precision in ith historical study
tau0[i] ~ dgamma(1e-3, 1e-3)
```

```
##Each observation in ith historical study
for (j in studyindex[2*(i-1)+1]: studyindex[2*i]) {
response_historical[j] ~ dnorm(mu0[j], tau0[i])
mu0[j] <- fixef_historical[j,] %*% beta_historical[i,1:num_fixef]
}
}
##Priors
##Overall mean of intercept and baseline effect
beta ~ dmnorm(mean_beta, prec_beta)
##Sigma_beta, between-study covariance matrix
sigma_beta0 ~ dnorm(0, prec_sigma_beta0) T(0,)
sigma_beta1 ~ dnorm(0, prec_sigma_beta1) T(0,)
rho_cor ~ dunif(-1, 0)
rho <- ifelse(cor, rho_cor, 0)
c <- 1/((1-rho^2)*(sigma_beta0^2*sigma_beta1^2))
Omega_beta[1, 1] <- c*sigma_beta1^2
Omega_beta[2, 2] <- c*sigma_beta0^2
Omega_beta[1, 2] <- -c*rho*sigma_beta0*sigma_beta1
Omega_beta[2, 1] <- Omega_beta[1, 2]
##Predicitve distribution for the new intercept and baseline effect
beta_new ~ dmnorm(beta, Omega_beta)
}
```

In addition, JAGS scripts for the analysis phase are shown as follows:

```
model {
##Historical likelihood
##Each of H historical studies
for (i in 1:H){
##Intercept and baseline effect in ith historical study
beta_historical[i,1:num_fixef] ~ dmnorm(beta, Omega_beta)
##Error precision in ith historical study
tau0[i] ~ dgamma(1e-3, 1e-3)
##Each observation in ith historical study
```

```
for (j in studyindex[2*(i-1)+1]: studyindex[2*i]) {
response_historical[j] ~ dnorm(mu0[j], tau0[i])
mu0[j] <- fixef_historical[j,] %*% beta_historical[i,1:num_fixef]
}
}
##Priors
##Overall mean of intercept and time effect
beta ~ dmnorm(mean_beta, prec_beta)
##Sigma_beta, between-study covariance matrix
sigma_beta0 ~ dnorm(0, prec_sigma_beta0) T(0,)
sigma_beta1 ~ dnorm(0, prec_sigma_beta1) T(0,)
rho_cor ~ dunif(-1, 0)
rho <- ifelse(cor, rho_cor, 0)
c <- 1/((1-rho^2)*(sigma_beta0^2*sigma_beta1^2))
Omega_beta[1, 1] <- c*sigma_beta1^2
Omega_beta[2, 2] <- c*sigma_beta0^2
Omega_beta[1, 2] <- -c*rho*sigma_beta0*sigma_beta1
Omega_beta[2, 1] <- Omega_beta[1, 2]
##Intercept and baseline effect in the new trial
beta_new ~ dmnorm(beta, Omega_beta)
##Treatment effect in the new trial
lambda ~ dnorm(0, 1e-4)
##Error precision in the new trial
tau ~ dgamma(1e-3, 1e-3)
##New trial likelihood
for (i in 1:N) {
response_new[i] ~ dnorm(mu[i], tau)
mu[i] <- fixef_new[i,] %*% c(beta_new, lambda)
}
}
```
